# The Future of Butyric Acid in Industry

**DOI:** 10.1100/2012/471417

**Published:** 2012-04-19

**Authors:** Mohammed Dwidar, Jae-Yeon Park, Robert J. Mitchell, Byoung-In Sang

**Affiliations:** ^1^School of Nano-Bioscience and Chemical Engineering, Ulsan National Institute of Science and Technology, Ulsan, Republic of Korea; ^2^New Renewable Energy Lab, SK Innovation Global Technology, Seoul, Republic of Korea; ^3^Department of Applied Chemical Engineering, Hanyang University, Seoul, Republic of Korea

## Abstract

In this paper, the different applications of butyric acid and its current and future production status are highlighted, with a particular emphasis on the biofuels industry. As such, this paper discusses different issues regarding butyric acid fermentations and provides suggestions for future improvements and their approaches.

## 1. Butyric Acid in Biofuels and Other Industrial Applications

Butyric acid has many uses in different industries, and currently there is a great interest in using it as a precursor to biofuels. Due to increases in petroleum prices as well as a continuous reduction in petroleum availability and a growing need for clean energy sources, research has recently been directed towards alternative fuel sources. Biofuels in general offer many advantages including sustainability, a reduction of greenhouse gas emissions, and security of supply. The term biofuel generally refers to solid, liquid, or gaseous fuels that are predominantly produced from biomass and will be used as such throughout this paper. Furthermore, liquid biofuels can be broadly classified into (a) bioalcohols, (b) vegetable oils and biodiesels, and (c) biocrude and synthetic oils [1].

As it is one of the most promising biofuels for replacing gasoline in the future, a lot of attention these days is paid toward biobutanol. Its primary use is as an industrial solvent, but it also offers several advantages over ethanol as a transportation fuel. For instance, the three carbon-carbon bonds in butanol provide more energy when burned than the two bonds present in two molecules of ethanol, that is, four carbons total for each fuel. In addition, butanol is less volatile than ethanol, can replace gasoline in internal combustion engines without any mechanical modifications, does not attract water like ethanol so it can be transported in existing pipelines, is not miscible with water, and is less sensitive to colder temperatures [2].

Despite these benefits, the fermentative bioproduction of butanol faces many problems as this alcohol is much more toxic to *Clostridia* than ethanol is to *Zymomonas mobilis*, which in turn results in lower concentrations in the fermentation broth, lower yields of butanol, and higher recovery costs. One feasible strategy to reduce the toxicity and improving the yield of butanol is to first ferment biomass into butyric acid and then convert this downstream into butanol [2]. In addition, butyric acid can be used to produce ethyl butyrate and butyl butyrate, both of which can be used as fuels [3].

In addition to its use as a biofuel, butyric acid has also many applications in pharmaceutical and chemical industries. Firstly, butyric acid is well known for its anticancer effects as it induces morphological and biochemical differentiation in a variety of cells leading to concomitant suppression of neoplastic properties [4–7]. Consequently, these studies present on various prodrugs that are derivatives of butyric acid were tried for their potential use in treatment of cancers and hemoglobinopathies, including leukemia and sickle cell anemia (SCA), and also to protect hair follicles of radio- and chemotherapy-induced alopecia. Butyrate also helps protect colonic mucosa from oxidative stress and inhibits its inflammation while promoting satiety [6]. In chemical industries, the main application of butyric acid is in the manufacture of cellulose acetate butyrate plastics [8]. By introducing the butyryl group into cellulose acetate polymers, the resulting polymer exhibits better performance in terms of its solubility in organic solvents due to enhanced hydrophobicity, better flexibility, and light and cold resistance [8]. A recent study also showed the possibility of mixing cellulose acetate butyrate (CAB) with another polymer, poly(3-hydroxybutyrate) (PHB), which can also be prepared from butyric acid [9], to decrease the production cost of PHB and improve its characteristics [10]. Furthermore, although butyric acid itself has an unpleasant odor, butyric acid esters such as methyl, ethyl, and amyl butyrate are used as fragrant and flavoring agents in beverages, foods and cosmetic industries [11, 12].

## 2. Current Status of Butyric Acid Production and Future Needs

Butyric acid is produced at the industrial scale via mainly a chemical synthesis. This involves the oxidation of butyraldehyde, which is obtained from propylene derived from crude oil by oxosynthesis [13]. The chemical synthesis of butyric acid is preferred mainly because of its lower production cost and the availability of the starting materials. Another method for butyric acid preparation is its extraction from butter. This is possible since its concentration in butter ranges from 2% to 4% but the process involved is difficult and expensive and, thus, cannot compete with the chemical alternative [14]. A third way is through fermentation. Although this method is currently more expensive compared to the chemical synthesis, it has garnered more attention due to both a growing consumer desire for organic and natural products, as opposed to chemically synthesized ingredients, and a continuous increase in the prices of crude oil, which is needed for the chemical synthesis as noted earlier.

## 3. Strains Available for the Bioproduction of Butyric Acid

Under anaerobic conditions, butyric acid is a common metabolite produced by bacteria strains from various genera. However, for industrial use, Clostridial strains are preferred owing to their higher productivities and the final concentrations obtained. The most important *Clostridium* strains studied for industrial scale productions of butyric acid are *C. butyricum *[15–17], *C. tyrobutyricum* [18–24], and *C. thermobutyricum *[25] (Table 1). Currently, the most promising microorganism used for the bioproduction of butyric acid is *C. tyrobutyricum*. This strain is capable of producing butyric acid with high selectivity and can tolerate high concentrations of this compound. However, it can only ferment very few carbohydrates, including glucose, xylose, fructose, and lactate, while its ability to utilize mannitol or glycerol is doubtful [26, 27]. On the other hand,* C. butyricum* can ferment many carbon sources including hexoses, pentoses, glycerol, lignocellulose, molasses, potato starch, and cheese-whey permeate [17]. However, compared to *C. tyrobutyricum*, the butyrate yields have always been much lower (Table 1). For *C. thermobutyricum*, the range of fermentable sugars is somewhat between these other two strains as it consumes glucose, fructose, maltose, xylose, ribose, and cellobiose, and some oligomeric and polymeric sugars but not sucrose or starch [28].

## 4. Butyric Acid Biosynthesis in *Clostridium* and Factors Affecting Its Production

**Figure 1:**
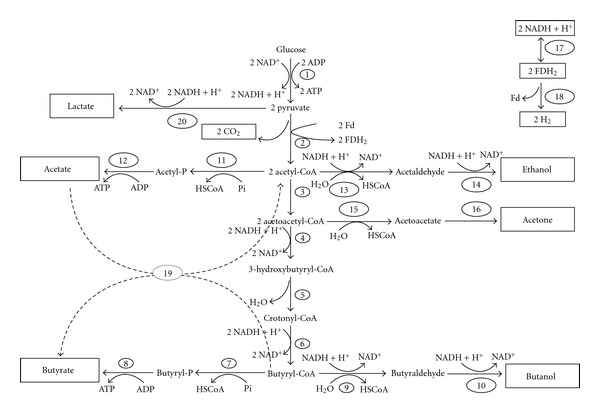
Metabolic pathway of butyrate production in Clostridia [19, 34, 35], 1: EMP pathway, 2: pyruvate-ferredoxin oxidoreductase, 3: acetyl CoA-acetyl transferase (thiolase), 4: *β*-hydroxy butyryl CoA dehydrogenase, 5: crotonase, 6: butyryl CoA dehydrogenase, 7: phosphotransbutyrylase, 8: butyrate kinase, 9: butyraldehyde deydrogenase, 10: butanol dehydrogenase, 11: phosphotransacetylase, 12: acetate kinase, 13: acetaldehyde dehydrogenase, 14: ethanol dehydrogenase, 15: CoA transferase, 16: acetoacetate decarboxylase, 17: ferredoxin-NAD(P)+ reductase, 18: hydrogenase, 19: butyryl CoA-acetate transferase (proposed enzyme), 20: lactate dehydrogenase.

For the fermentative process to proceed, glucose must first be converted to pyruvate via the Embden-Meyerhof-Parnas (EMP) pathway, which produces two moles of ATP and NADH. Subsequently, pyruvate is fermented to produce several products, depending on the strain. Lactate dehydrogenase catalyzes the conversion of pyruvate to lactate while pyruvate-ferredoxin oxidorecuctase catalyzes its conversion to acetyl-CoA with the release of CO_2_ and the generation of reduced ferredoxin (fdH_2_). For acetate production, phosphotransacetylase (PTA) and acetate kinase (AK) are the key enzymes while, for butyrate, phosphotransbutyrylase (PTB) and butyrate kinase (BK) play similar roles (Figure 1). During acetate production, 4 moles of ATP are formed, while during butyrate production only 3 moles of ATP are formed, which helps to explain why, at high growth rates, cells shift more towards acetate rather than butyrate production [32, 33]. At the end of the exponential growth, the organisms slow down acetate production and take up excreted acetate, converting it into butyrate. This recycling may be an attempt of the organism to detoxify the medium by reducing the total hydrogen ion concentration, that is, acid concentration, as one butyric acid is produced from two acetic acids. Consequently, the metabolism is shifted from the more energy conserving acetate formation (1) to a lower acid content with butyrate formation (2) [9]:

(1) $$\text{Glucose}\to 2\;\text{Acetate}+4{\text{H}}_{2}+2\text{C}{\text{O}}_{2}+4\text{ATP}$$

(2) $$\text{Glucose}\to \text{Butyrate}+2{\text{H}}_{2}+2\text{C}{\text{O}}_{2}+3\text{ATP}.$$

For those strains capable of producing solvents (butanol and acetone), the fermentation usually passes through two steps—the acidogenesis phase in which butyric and acetic acids are both produced in the medium and then the solventogenesis phase in which the organism converts these acids into acetone, ethanol, and butanol [14]. This second stage is initiated as the medium becomes more acidic and the cells enter the stationary phase [14].

### 4.1. Factors Affecting Butyrate Production and the Acetate/Butyrate Ratio

There are many factors that can affect the production characteristics. In general, butyrate production is higher in fed-batch, glucose-limited, and slow-growing cultures than in classic batch cultures [32, 36]. Likewise, the addition of acetate can significantly change the final butyrate concentrations. For example, it was found that the addition of acetate to cultures of *C. thermobutyricum* leads to higher final optical densities, a higher consumption of glucose, and faster growth rates [37]. Furthermore, in another study done recently [33], the addition of acetate (10 g/L) to a continuous culture of *C. thermobutyricum* led to 20% increase in the average concentration of butyric acid in the culture (from 10.23 g/L to 12.27 g/L). A similar phenomenon also was observed for *C. tyrobutyricum* CNRZ 59 [38]. Also for *C. beijerinckii *NCIMB 8052, it was found that cultures grown in medium containing added acetate exhibited higher CoA transferase, acetate kinase, and butyrate kinase activities than cultures grown in its absence [39].

The nutritional value of the medium may also have a great effect on the productivity and final butyrate concentration. Usually, butyric acid production is enhanced in rich medium. For *C. tyrobutyricum*, it gave more butyric acid in RCM media (which has more yeast extract and tryptone) compared to the Clostridial growth media (CGM) [23]. For *C. thermobutyricum*, it was found that its growth and glucose utilization is dependent on the yeast extract concentration [37]. Furthermore, peptone or tryptone was not adequate substitute for yeast extract and their addition to a 0.5% glucose-containing medium (to substitute for yeast extract) caused a strong increase in the frequency of sporulation. Yeast extract is also necessary for the growth of the butyrate-producing thermophile *C. thermopalmarium* [40]. These effects of yeast extract are common among other Clostridia [41] but are not so surprising as yeast extract is a complex nutritional source consisting of amino nitrogen, vitamins and other unknown growth factors used by many microbial organisms [42].

Trace elements were found also to affect butyrate production, especially iron and phosphate. For *C. butyricum* grown on glycerol as a carbon source, it was found that phosphate limitation caused an increase in butyrate yield and a decrease in acetate yield, leading to a diminished acetate/butyrate ratio while iron limitation caused the reverse [43]. Interestingly, however, both resulted in significant improvement in the 1,3-propandiol production and a decrease in the H_2_/CO_2_ ratio. The authors assumed that the hydrogenase enzyme (the enzyme responsible for releasing hydrogen from reduced ferredoxin instead of storing it as NAD(P)H_2_) of *C. butyricum* contains iron and that it is greatly affected by iron limitations. They claimed that iron and phosphate limitation results in greater availability of reducing equivalents (NADH_2_) and this in turn favors the production of NADH-dependent products, that is, mainly 1,3 propanediol (which has higher capacity for NADH consumption) and to a lesser extent butyrate and ethanol. However, with regard to the butyrate pathway, an iron limitation probably results in a reduced butyrate kinase activity and, thus, inhibition of butyrate production [44].

A similar effect to phosphate limitation can be also achieved by the addition of a suitable electron mediator, such as methyl viologen, which can act as an artificial electron-carrier system like ferredoxin, accepting electrons coming from pyruvate-ferredoxin oxidoreductase. As the electrons bound to methyl viologen are less available for the hydrogenase than those coming from ferredoxin, a greater proportion of electrons are channeled to the ferredoxin-NAD(P) reductase to produce NAD(P)H_2_ and thus the acetate/butyrate ratio will decrease [43, 45]. The activity of the hydrogenase can be also decreased through an increase in the hydrogen partial pressure [36, 43, 46] or through carbon monoxide flushing (a reversible inhibitor of hydrogenase) [47]. In contrast to these studies, when Jo et al. [48] overexpressed the [Fe Fe]-hydrogenase in *Clostridium tyrobutyricum* JM1, their purpose being to enhance hydrogen production, they found that significant changes in the main metabolic pathways occurred, which led to an improved butyric acid yield while also suppressing lactic acid production [48]. Therefore, it seems that hydrogenases are key enzymes controlling the butyric, acetic, and lactic acids pathways and that their activities should be carefully tuned or monitored to achieve an optimum production.

### 4.2. Effect of Carbon Source and Fermentation Products

In general, butyric acid fermentations are more prolific in terms of their yields, productivity, and final butyrate concentrations when performed with limited glucose concentrations as compared to those done with excess glucose, as the latter often leads to osmotic dehydration of the cells [49]. However, end product inhibition is the main problem that faces butyric acid fermentations, and different approaches are being tried to improve the tolerance of *Clostridium* strains to the produced butyric acid. Butyric acid can pass through the bacterial membrane in its unionized form and then dissociates inside the cell leading to disturbance in the pH gradient across the cell membrane and thus more energy is consumed to restore and maintain a functional pH gradient across the membrane, which in turn will limit the energy available for biomass growth [38, 50].

It was observed that butyric acid produced by the culture is more toxic than that externally added, and the same seems to apply for acetic acid. This could be explained by the fact that acid concentration, within acid-producing cells, is higher than that when the acids are added externally [51]. Butyrate was found to cause more inhibition compared to acetate for *C. tyrobutyricum* and also for *C. acetobutylicum*, *C. populeti*, and *C. thermocellum *[32]. In general, the inhibitory effects of the byproducts (e.g., acetate, butanol, ethanol, and acetone) appear in a concentration range that is above the concentrations usually reached during butyric acid fermentations. For solventogenic strains, which convert the produced butyrate into butanol, butanol is the toxic end product and negatively impacts the culture through its fluidizing effect on the lipids of the cell membrane, leading to disruption of the membrane function [52].

## 5. Approaches for Improving Butyric Acid Bioproduction

### 5.1. Fermentation Approaches

Almost all known fermentation modes, ranging from batch and fed-batch to continuous fermentations with and without cell recycling, were evaluated during butyrate production by different research groups. For fed-batch fermentation, the rate of substrate supply is a key factor for optimizing the production. In 1989, Fayolle et al. reported that substrate feeding controlled by the rate of gas production was preferable to a constant rate feeding [18].

Compared to batch and fed-batch modes, continuous cultures can give higher productivities but lower product concentration. To enhance the productivity even more, cell recycling systems can be applied with a continuous process design, allowing operation at a high cell concentration and at a limited growth rate, and, consequently, a high butyrate production rate and selectivity can be achieved [24, 32, 38]. Another way to maintain higher cell density and thus higher butyrate productivity is through cell immobilization [14, 53], but it should be kept in mind that immobilized cell bioreactors tend to lose their productivity over time due to the accumulation of old inactive cells. This drawback can be observed in various kinds of cell immobilization techniques [9]. However, fibrous bed bioreactors (FBBs) seem to be less affected by this phenomenon as compared to other systems and can maintain their productivity over a longer period of time if the operative conditions are carefully controlled [54]. This is attributed to their large void volume and high permeability, which give them regenerative ability and high mass transfer capabilities [20]. It should not come as a surprise, therefore, that FBBs have been successfully used by different research groups to produce butyric acid [20, 33, 55, 56].

#### 5.1.1. In Situ (Online) Product Removal

Online product removal through different techniques including dialysis, distillation, adsorption, and extraction was tried for isolation of carboxylic acids and other volatile products, and many of them can be applied for butyric acid fermentations [57]. In situ removal of the product can improve the fermentation process and enhances the productivity by decreasing the concentration of this product in the culturing medium and therefore reducing its toxic effect on the cells. Besides, this greatly reduces the need for the tedious and time consuming separation steps that follow the fermentation process.

For electrodialysis, the biomass should be separated first from the fermentation broth through micro- or ultrafilteration, and then the product of interest can be subsequently separated through permeate electrodialysis. This method was applied successfully for the separation of propionic, lactic, and acetic acids [58–60]. Pervaporation processes are also frequently used for isolation of volatile products, and they were successfully used for the in situ removal of solvents in ABE fermentations using *C. acetobutylicum* as a fermenting strain [61]. Also adsorption is fairly common for the isolation of fermentation products such as butanol using various materials as adsorbents [62, 63]. The use of extraction and pertraction methods was mentioned by many research groups for the isolation of products like organic acids and other fermentation products; however, several factors have to be considered before the butyrate fermentation is coupled with extraction or pertraction. Both physical and reactive extractions have been described and evaluated for their potential application in connection with fermentation. Reactive extraction in general has higher efficiency compared to the physical one because the organic phase also contains a reactant or carrier. It means that the acid is extracted into an organic phase by physical transport and complexation with the carrier. Several chemicals can be applied as the organic phase but the toxicity of the organic solvent that can be estimated from its Log *P* (partition coefficient) value must be considered first together with its extractive ability [64]. One way to decrease this toxicity is through immobilizing the cells and thus protecting them from being in close contact with the organic solvent [22]. Further protection can be achieved also through adding vegetable oils such as castor, soy bean, and olive oils to the immobilization matrix to trap the diffusing solvent molecules in the fermentation medium [65]. For example, some authors reported the use of a mixture of Al_2_O_3_ and sunflower oil to decrease the toxicity of decanol in extractive fermentation for ethanol production using *S. cerevisiae* cells immobilized in calcium alginate gel, and they claimed the possibility of applying similar strategies for other valuable chemicals also [66]. Similarly, another research group reported the possibility of decreasing the toxicity of alamine 336/oleyl-alcohol extraction system on *Lactobacillus delbrueckii* through immobilization and adding soybean oil to the *κ*-carrageenan matrix [67]. The effectiveness of the extraction process and the distribution coefficient depend greatly on pH as the organic solvents can extract only undissociated acids. This problem can be partially solved by reactive extraction or by pertraction in which the product is extracted from the fermentation broth and simultaneously stripped from the organic phase into the stripping solution. The organic phase is simultaneously regenerated in this process.

Extraction and pertraction were used effectively for acetate, propionate, and lactate fermentations and also for ABE fermentation. For butyrate fermentation, Zigova et al. in 1996 [68] tested different solvents including tertiary amines, alcohols, alkanes, and vegetable oils for their potential use as extractants, and they found that solvents with tertiary amines were the best in terms of their butyric acid extractive abilities. The toxicity also of various solvents for *C. butyricum* was evaluated by the same research group [69]. In 1999, they showed the possibility of integrating extraction and pertraction methods with fermentative production of butyric acid by *C. butyricum*. They used Hostarex (20% w:w) in oleylalcohol as an extractant, and the concentration of butyric acid produced was increased from 7.3 g/L in the control to 10.0 g/L in extractive fermentation and to 20.0 g/L in pertractive fermentation with concurrent increases in the yield also [16]. In 2003, Wu and Yang reported also on the use of pertractive fermentation for butyric acid production from glucose, using immobilized cells of *C. tyrobutyricum *in a fibrous bed bioreactor. In their study, they used 10% (v/v) alamine 336 in oleyl alcohol as the extractant with simultaneous regeneration through continuous stripping with NaOH [22].

Recently, another research group reported on the use of a tertiary alkyl phosphine compound, tri-n-octylphosphine oxide (TOPO) (which is more environmentally friendly than amines) dissolved in seven different solvents for extraction of proionic and butyric acids from aqueous solutions [70]. It was observed that the use of TOPO dissolved in these diluents increased the distribution coefficients of propionic and butyric acids between the organic and aqueous phases up to 51.22 and 12 times, respectively. The results also showed that the highest distribution coefficient for both acids is obtained by TOPO dissolved in methyl tert-butyl ether (MTBE). It should be noted, however, that the extraction ability of different organic mixtures is highly affected by temperature [71].

Aside from the use of extraction in online butyric acid removal, it can still be used also off line for downstream purification of butyric acid from the fermentation broth. Wu et al. [3] reported on the possibility of selective extraction of butyric acid from the fermentation broth through salting out using inorganic salts as calcium chloride [3]. This “salting out” effect was very efficient to separate butyric acid from the simulated butyrate fermentation broth (which consisted of butyric acid and acetic acid in a concentration ratio of 4 : 1) so that the final ratio of butyric acid/acetic acid in the upper phase was 9.87 : 1. 

Some authors also suggested a novel approach for selective separation of dilute products from simulated *Clostridium *fermentation broth through the application of cyclodextrins. The ability of cyclodextrins (CDs) to form crystalline insoluble complexes with specific organic compounds was used to separate the ABE fermentation products together with acetic and butyric acids separately. Cyclodextrins were thus shown to offer a new exciting possibility for downstream processing of those products [72].

As the production of butyric acid and carboxylic acids in general is usually done near the neutral pH and in the form of salts, which requires the addition of mineral acids after finishing the fermentaion for the separation of the free acid, some authors proposed an alternative approach for carboxylic acids production. This approach is through two-stage fermentation process in which the fermentation medium containing a lactate salt is first fermented with the appropriate strain to give the salt of the required acid and then a fermentable carbohydrate is added into the medium to make the second fermentation medium which can be fermented by a lactobacillus strain under conditions suitable for converting the carbohydrate to a lactate salt while converting the salt of the selected acid into the free acid, then separating this free acid and recycling the medium again [73].

### 5.2. Approaches for Strain Improvement

Conventional butyric acid fermentation process is not yet economically competitive as the final concentration of butyric acid in the fermentation broth is still much lower than the required values with low yield as well as low productivity. Besides, acetic acid is produced as a byproduct in the process that causes further reduction in butyrate yield and increases product recovery and purification costs. To improve the economics of the fermentation process, it is desirable to increase butyrate while reducing acetate production [9]. To increase butyrate production, genetic methods were applied to *C. tyrobutyricum* ATCC 25755 strain aiming to delete the acetate-producing pathway [21]. The mutants of *C. tyrobutyricum* with either *pta* (phosphotransacetylase) or *ak* (acetate kinase) genes knocked out were constructed. Compared with the wild type, butyric acid production by these mutants was improved with higher final product concentration and yield. Also, these mutants have better tolerance to butyric acid inhibition. The ack-deleted mutant also has improved hydrogen production. However, acetic acid production in these mutants was not significantly affected in the fermentations even though the mutants had a much lower AK activity and the PTA-AK pathway should have been impaired. Besides PTA and AK, the authors suggested the presence of other enzymes in *C. tyrobutyricum*, which can also produce acetate, for example, CoA transferase, which can catalyze the formation of acetate from acetyl-CoA [21].

Sillers et al. [74] also reported on the metabolic engineering of the nonsporulating, nonsolvent-producing *C. acetobutylicum* M5 strain (which has lost the pSOL1 megaplasmid containing *aad* and the acetone formation genes) [74]. In that study, they tried to combine *thl* (thiolase) overexpression with *aad* (alcohol/aldehyde dehydrogenase) overexpression aiming at enhancing butanol formation. While this approach succeeded in limiting the formation of acetate and ethanol, the butanol titers were not improved. However, they found that overexpressing *thl* led to significant increase in butyric acid production.

Recently, Jiang et al. [56] reported on the adaption of *C. tyrobutyricum* in immobilized cultures in an FBB [56]. This adaptation resulted in significantly reduced inhibition effects of butyric acid on specific growth rate and also on cellular activities of phosphotransbutyrylase (PTB) and ATPase, together with elevated intracellular pH. Using this adapted strain, they could reach a final butyric acid concentration of about 86.9 g/L. This may be further improved in the future if the molecular mechanisms leading to growth inhibition by butyric acid can be determined.

## 6. Summary and Future Research

Nowadays, there is a great need for butyric acid produced by microbial fermentation in various industries. The main future challenge for this will depend upon increasing butyrate tolerance of Clostridia strains and decreasing the cost of culture media materials. Lignocellulosic materials have the potential to serve as a cost-effective source of raw material for the production of liquid fuels, including bioethanol and biobutanol, and various organic chemicals. The main obstacle that faces the use of lignocellulosic feedstocks for fuel production is the high cost of hydrolyzing cellulose into simple monomeric sugars.

One suggested approach for solving this problem is through coculturing a strain capable of degrading these carbohydrate polymers such as cellulose, hemicelluloses, and starch with a *Closteridium* strain capable of producing the biofuel or the carboxylic acid of interest [9]. For example, it may be possible to coculture *C. thermocellum* and *C. thermobutyricum* for the production of butyric acid using cellulose as a carbon source. *C. thermocellum* is capable of producing cellulase and hemicellulase and is capable of utilizing the hexoses, but not the pentose sugars, generated from cellulose and hemicellulose. Although the main secondary metabolite from *C. thermocellum* is ethanol, not butyric acid, it suffers from a low growth rate [41], allowing *C. thermobutyricum*, which ferments pentose and hexose sugars more quickly than *C. thermocellum*, to utilize the sugars liberated and generate butyric acid. The recombinant technology to develop a cellulolytic organism is also possible, especially for *C. thermobutyricum* as its culture temperature is similar to the temperature at which cellulase is most active [9]. On the other hand, Chang et al. [75] reported that the combination of aerobic *Bacillus* and anaerobic *Clostridium* may play the key role in the future of biofuel production from biomass since strains of *Bacillus* have high growth rates in general and they can secrete many extracellular sacchrification enzymes in the medium such as amylase, pectinase, protease, cellulase, and hemicellulase. Tran et al. [76] also have shown recently the potential of coculturing *B. subtilis* with *C. botulinum* for butanol production from starch [76]. Besides those ordinary carbon sources, other groups suggested also the possible use of substrates as alcohols for carboxylic acid production through biotransformation using strains as *Acetobacter aceti* (for butyric acid from butanol) [77] and *Gluconobacter oxydans *(for propionic acid from n-propanol) [78].

Butyric acid is a promising chemical that may hold the potential for tomorrow energy needs as it can be converted to butanol through biological transformation. Even with the final goal being a higher butanol productivity, strains producing butyric acid may hold the key as they are capable of producing 3~5 times more butyrate than the current maximum seen for butanol, for example, about 20 g/L. Consequently, these strains can be used to generate a surplus of butyric acid, which can be converted downstream as needed.

## Figures and Tables

**Table 1:** Summary of the most promising butyrate producing clostridial strains.

Strain	Sugar	Final conc. (g/L)	Fermentation mode	Reference
C. *butyricum *ZJUCB	Glucose	12.25	Batch	[17]
		16.74	Fed-batch	
*C. butyricum *S21	Glucose	7.3	Batch	
	Sucrose	10	Extractive batch	[16]
	Sucrose	20	Pertractive fed-batch	
*C. butyricum *S21	Lactose	18.6	Batch	[13]
*C. thermobutyricum *ATCC 49875	Glucose	10.04	Batch	[25]
		19.38	Continuous	
*C. beijerinckii*	Lactose	12	Batch	[29]
*C. populeti* *ATCC 35295 *	Glucose	6.3	Batch	[30]
*C. tyrobutyricum *JM1	Glucose	13.76	Batch	[19]
*C. tyrobutyricum *CIP 1–776	Glucose	45	Batch	[18]
	Glucose	62.8	Fed-batch	
	Glucose	28.6	Fed-batch	[21]
*C. tyrobutyricum *ATCC 25755	Glucose	24.88	Fed-batch (immobilized cells)	[20]
	Glucose	43.4	Fed-batch (immobilized cells)	[22]
	Glucose	53	Fed-batch (immobilized cells)	[23]
*C. tyrobutyricum *CNRZ 596	Glucose	44	Batch	
	Glucose	16.8	Continuous	[24]
	Glucose	33	Continuous (cell recycle)	
*C. tyrobutyricum *ZJU 8235	Jerusalem artichoke hydrolysate	27.5 g/L	Batch	[31]
		60.4 g/L	Fed-batch (immobilized cells)	
				
